# An improved procedure for isolating adult mouse cardiomyocytes for epicardial activation mapping

**DOI:** 10.1111/jcmm.17049

**Published:** 2021-11-10

**Authors:** Ziguan Zhang, Wuyang Zheng, Dehua He, Zichao Hu, Qiang Xie, Meirong Huang, Weihua Li, Zhengrong Huang

**Affiliations:** ^1^ Department of Cardiology Xiamen Key Laboratory of Cardiac Electrophysiology Xiamen Institute of Cardiovascular Diseases The First Affiliated Hospital of Xiamen University School of Medicine Xiamen University Xiamen Fujian China; ^2^ Technical Support Department Henan SCOPE Research Institute of Electrophysiology Co. Ltd Kaifeng Henan China; ^3^ Department of Echocardiography The First Affiliated Hospital of Xiamen University Xiamen Fujian China

**Keywords:** adult mouse cardiomyocytes, cardiomyocyte isolation, electrophysiology, epicardial activation mapping, Langendorff

## Abstract

Cardiovascular disease is a leading cause of death and disability worldwide. Although genetically modified mouse models offer great potential for robust research *in vivo*, *in vitro* studies using isolated cardiomyocytes also provide an important approach for investigating the mechanisms underlying cardiovascular disease pathogenesis and drug actions. Currently, isolation of mouse adult cardiomyocytes often relies on aortic retrograde intubation under a stereoscopic microscope, which poses considerable technical barriers and requires extensive training. Although a simplified, Langendorff‐free method has been used to isolate viable cardiomyocytes from the adult mouse heart, the system requires enzymatic digestions and continuous manual technical operation. This study established an optimized approach that allows isolation of adult mouse cardiomyocytes and epicardial activation mapping of mouse hearts using a Langendorff device. We used retrograde puncture through the abdominal aorta *in vivo* and enzymatic digestion on the Langendorff perfusion device to isolate adult mouse cardiomyocytes without using a microscope. The yields of isolated cardiomyocytes were amenable to patch clamp techniques. Furthermore, this approach allowed epicardial activation mapping. We used a novel, simplified method to isolate viable cardiomyocytes from adult mouse hearts and to map epicardial activation. This novel approach could be beneficial in more extensive research in the cardiac field.

## 1 INTRODUCTION

Cardiomyocytes are terminally differentiated cells and thus cannot proliferate in response to injury and death. Although some atrial cardiac cell lines, such as AT‐1 and HL‐1,^1^ and human ventricular cardiac cell lines, such as AC16,^2^ have been used in *in vitro* studies, their characteristics are not identical to mature cardiomyocytes.^3^ Therefore, these cell lines are not ideal for studying cardiomyocyte physiology and pathophysiology *in vitro*.

Currently, cardiomyocytes are often isolated from adult mice through retrograde aortic perfusion for *in vitro* drug screening.^4^ However, the mouse aorta is small, and retrograde perfusion requires a stereoscopic microscope and must be performed by trained researchers, which poses a technical barrier for many laboratories. One previous study^5^ suggested that myocardial cells can be successfully isolated by enzymatic digestion *in vitro* without Langendorff equipment. However, this method requires various enzymatic digestions, continuous manual technical operation and does not allow for epicardial activation mapping.

In this study, we describe a simplified retrograde aortic intubation technique using the Langendorff device to isolate adult mouse cardiomyocytes. Our method greatly reduces the difficulty of operation, shortens the time for connecting the aorta to the Langendorff device, and subsequently isolates more living cardiomyocytes. We expect that this approach will be extensively used in the cardiac field.

## 2 MATERIALS AND METHODS

### 2.1 Animals and ethics

Animal experiments were conducted according to the procedures and guidelines of Xiamen University, and the protocols were approved by the Institutional Animal Care and Use Committee of Xiamen University, College of Medicine.

### 2.2 Perfusion system preparation

The perfusion system was flushed with sterile deionized water for 1 h to keep the system clean. The flow speed was adjusted to obtain a perfusion rate of 2–4 ml/min. The temperature of the circulating water was maintained at 37℃ ± 0.5℃. Air bubbles in the system were removed to ensure a smooth flow of perfusion fluid through the whole tube. The perfusion fluid was saturated with medical oxygen at a purity of more than 99% (95% O_2_ + 5% CO_2_ for mapping) for more than 30 min.

### 2.3 Preparation of culture media, buffers and dishes

Perfusion buffer: We made 500 ml of 1x perfusion buffer prior to use. Instructions for making the buffers are provided below.

#### 2.3.1 *Tyrode solution (in mmol*/*L)*

NaCl 135, KCl 5.4, CaCl_2_ 1.8, NaH_2_PO_4_ 0.33, MgCl_2_ 1.0, HEPES 10, D‐glucose 10. Adjust the pH to 7.3 with 2 M NaOH, filter through a 0.2 μm filter and store at 4°C.

#### 2.3.2 *Calcium*‐*free tyrode buffer (in mmol*/*L)*

NaCl 135, KCl 5.4, NaH_2_PO_4_ 0.33, MgCl_2_ 1.0, HEPES 10, D‐glucose 10. Adjust the pH to 7.3 with 2 M NaOH, filter through a 0.2 μm filter and store at 4°C.

#### 2.3.3 *KH solution (in mmol*/*L)*

NaCl 118.5, NaHCO_3_ 25.0, KCl 4, MgCl_2_ 1, KH_2_PO_4_ 1.2, D‐glucose 10, CaCl_2_ 1.8. Gas with 95%O_2_ + 5%CO_2_ (pH 7.4). Filter through a 0.2 μm filter and store at 4°C.

### 2.4 Note

The perfusion solution was prepared prior to use. However, the perfusion solution may be stored at 4°C for no more than 3 days.

Digestion buffer (35–50 U/ml, 60–90 ml/heart): This buffer was prepared just prior to perfusion.

Dissolve 2000–2500 U of type II collagenase (9–10 mg), 3–4 mg protease E, and 30 mg bovine serum albumin (BSA) in 60 ml calcium‐free tyrode buffer. Add 60 μl 20 mmol/L CaCl_2_ every 5 min (stock solution, filter sterilized).

Stop buffer (50 ml/heart): Mix 50 ml of perfusion buffer with 50 mg BSA. Add 600 μl of 20 mM CaCl_2_ to 50 ml of stop buffer and mix. 10 min later, add another 600 ul of 20 mM CaCl_2_ to 50 ml of stop buffer and mix. 10 min later, add 800 μl of 20 mM CaCl_2_ to 50 ml of stop buffer and mix.

Note: Drip the buffer along the wall of the beaker slowly to avoid impacting the cells.

### 2.5 Mouse heart removal and cannulation

Scissors and forceps were soaked in 70% ethanol for 30 min. Heparin (200 ul, 100 IU/20 g) was intraperitoneally (i.p.) injected 10 min prior to anaesthesia to prevent coagulation in coronary arteries. The mouse was anaesthetized with 0.1 ml/10 g of 3% chloral hydrate i.p.; we waited 5–10 min until the mouse showed no response to tail/toe pinches. The mouse was fixed in the supine position on a work surface or station. We disinfected the local skin by spraying alcohol and cut open the abdominal skin. The abdominal skin was lifted with forceps, the chest skin was cut open to expose the xiphoid, and then, the xiphoid was lifted to cut open along the left and right costal ribs to expose the heart. We cut open the diaphragm (being careful not to damage the aorta) from the horizontal dissociation of the abdominal aorta of the lower limbs to the thoracic aorta section. A suture line (No.6) was placed under the abdominal aorta of the upper abdomen, and the abdominal aorta was punctured with an indwelling needle (No. 18) to the level of the heart root, and then, a suture line was placed. After emptying the gas bubbles, the heart was quickly inserted into the Langendorff equipment, which was perfused in advance. The entire procedure takes approximately 3 min (Figure S1 and video), which represents a significant improvement over other methods, such as retrograde perfusion, that require a stereoscopic microscope.

Note: (a) The perfusion fluid should be dripping during the cannulation procedure to prevent gas bubbles from entering the coronary arteries; (b) The tip of the cannula should be maintained inside the aorta; and (c) The full cannulation procedure should be completed in less than 2 min.

### 2.6 Digestion

Washing: The heart was flushed with 100 ml tyrode solution to wash out the remaining blood for 5 min. The heart was perfused with calcium‐free tyrode solution (100 ml contain EGTA 20 mg) to stop the heart beating.

Digestion: The heart was then perfused with 60 ml calcium‐free tyrode solution (collagenase Ι 9–10 mg, protease E for 3–5 mg, BSA 30 mg for mouse heart), and an additional 60 ul of 20 mmol/L CaCl_2_ every 5 min for 3–4 times. When the heart became semitransparent, distensible and puffy, and the enzymatic droplet that flows down appeared sticky, the digestion process was stopped. The total digestion time was 30~35 min. The flow rate for digestion was 8–12 ml/min.

Hatching: The ventricle was cut open and placed in a beaker containing the cell preservation solution (50 ml calcium‐free tyrode buffer containing 50 mg BSA). The ventricle was then finely chopped with scissors, stirred gently with a magnetic stirrer for 30 s, and diluted with cell preservation solution. The heart was incubated at 36°C~37°C for 5 min and rested for 10 min. The supernatant was removed, diluted with another 50 ml fresh calcium‐free tyrode solution (containing 50 mg BSA), then gently stirred with a magnetic stirrer for 30 s, incubated for 5 min, and set aside for 10 min.

Calcium was gradually restored. CaCl_2_ was added into the supernatant in three gradient stages: 600 ul, 600 ul and 800 ul of CaCl_2_ (20 mmol/L) were added at an interval of 10 min to reach the final concentration of 0.8 mmol/L. The cells were then stored at room temperature for about 1 h for subsequent experiments.

Counting method of calcium‐tolerant ventricular myocytes: The separated cardiomyocytes were stored at room temperature for about 1 h and were then placed into another bathing solution with a physiological calcium concentration. The standard calcium‐resistant cardiomyocytes were indicated by the following physiological characteristics: long rod‐shaped with sharp and clear edges, clear surface texture without vacuoles, and quiescent without spontaneous contraction.

The full isolation procedure is shown in a video provided in supplemental Figure 1.

**FIGURE 1:**
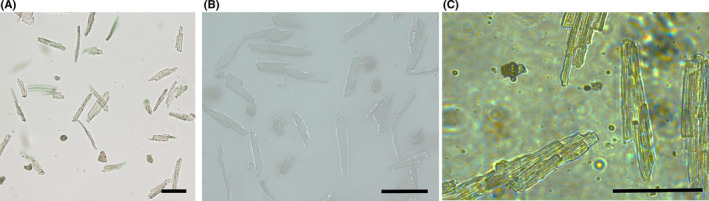
Isolation of adult mouse cardiomyocytes using the Langendorff device. Isolated cardiomyocytes display a long rod shape with clear horizontal stripes and sharp edges and are calcium tolerant (i.e. stay quiescent in tyrode solution). Scale bar =100 μm

### 2.7 Drug screen and mapping

The stimulating electrode was placed on the apex of the heart, and the left ventricular epicardium was contracted using the MappingLab matrix multi‐channel electrode. The ECG electrodes were placed in the right atrium and left ventricle of the heart.

After the heart was stabilized, signals were recorded using the MappingLab matrix multi‐channel electrophysiological mapping system (Baseline). To determine the pacing threshold of the diastolic period, a series of stimulations were applied, and the current intensity was slowly increased (the time interval was 2 ms). Diastolic threshold current intensity was determined as an obvious 1:1 conduction according to the set stimulus frequency, and the pacing current intensity was set as 2x the diastolic threshold current intensity.

### 2.8 Electrophysiological studies

Action potential, transient outward potassium current (Ito), L‐type calcium current (ICa,_L_) and sodium current (INa) were recorded at room temperature (22°C–25°C) using a Multiclamp 200B amplifier (Axon Instruments, Sunnyvale, CA). Patch pipettes (tip resistance was 2–3MΩ) were filled with the following solutions. The components of the pipette and bath solutions of the action potential, INa, Ito and ICa,_L_ were described previously.^6^, ^7^, ^8^ All products were purchased from Sigma (Madison, WI, USA). Junction potential, capacitance and series resistance were automatically compensated in the whole‐cell configuration. The holding potential and the voltage clamp were operated as described.^6^, ^7^, ^8^ The currents were filtered at 5 kHz, sampled at 50 kHz and stored on a desktop computer equipped with an AD converter (Digidata 1550B, Molecular Devices). All current measurements were normalized using the cell capacitance. Clampfit 10.2 (Axon Instruments) and Excel (Microsoft) were used for data acquisition and analysis.

### 2.9 Data analysis

Data are presented as the mean ±standard error of the mean (SEM). SSPS 17.0 was used for statistical analyses. Student's two‐tailed t tests were used for comparison of two group data. Statistical significance was set at the P level of <0.05.

## 3 RESULTS

### 3.1 Isolation of cardiomyocytes from adult mouse hearts using the Langendorff device

We improved the traditional method of Langendorff perfusion by retrograde abdominal aorta puncture using a trocar puncture. Cannulation and retrograde perfusion of the aorta promoted dissociation buffers to enter deep into the myocardium through the coronary vasculature. The matching trocar could be selected according to the age of mice, which greatly reduced the operative complexity of aortic intubation and greatly increased the success rate. Isolated cardiomyocytes are shown in Figure 1A–C and Supplemental Figure 2, which also shows isolated cardiomyocytes expressing GFP following adenovirus transfection. The crude digestion product was monitored in each experiment, and the total number and percentage of rod‐shaped viable cardiomyocytes were counted using a hemocytometer. After calcium reintroduction, cardiomyocytes remained quiescent, displayed a long rod shape and had clear horizontal stripes and sharp edges. With the conditions mentioned above, the protocol reproducibly yielded nearly 1 million cardiomyocytes per left ventricle, and 85%–92% of these cells were rod‐shaped viable cells. This was in agreement with yields previously reported using other isolation methods.^9^, ^10^

**FIGURE 2:**
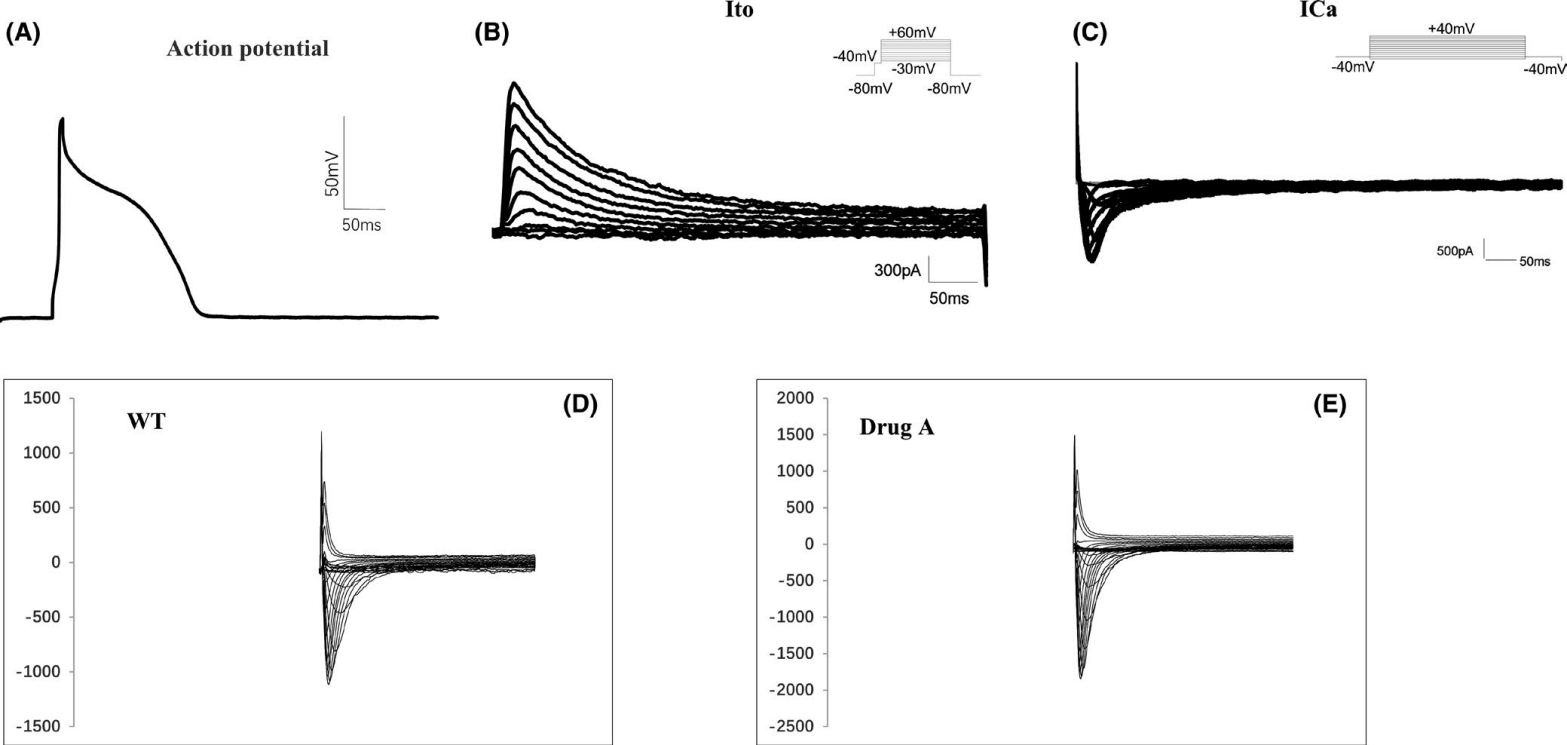
Electrophysiological characteristics of isolated cardiomyocytes indicated that the cells are amenable to patch clamp. (A) Ito current. (B) ICa, L current. (C) Action potential. (D) INa current. E. Influence on INa current by drug A

### 3.2 Electrophysiological characteristics of isolated cardiomyocytes by patch clamp

**FIGURE 3:**
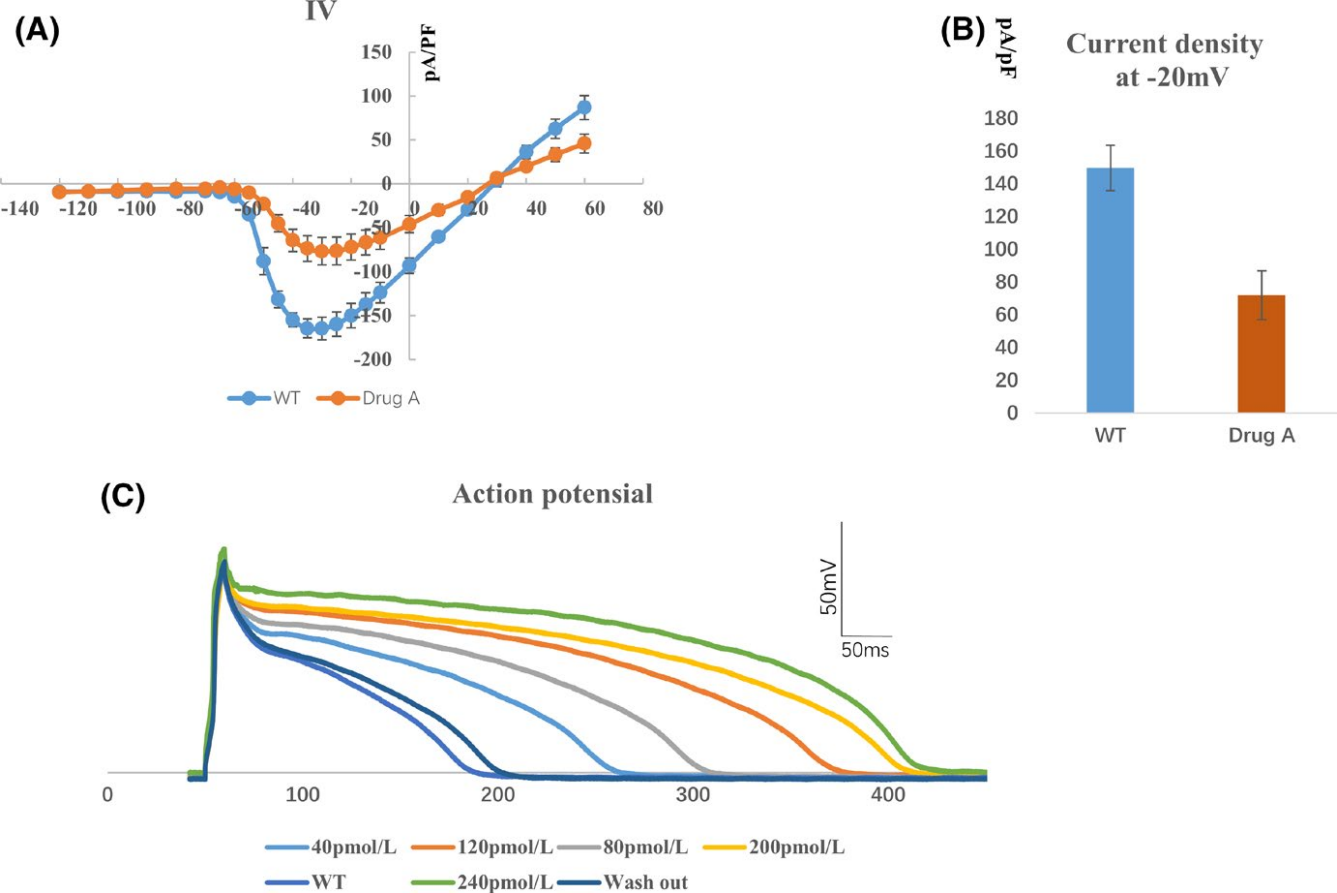
Effects of drug A on electrophysiological characteristics of isolated cardiomyocytes indicated that the cells are amenable to patch clamp. (A) I‐V curve of INa current. Average Na sodium current density is greater after treatment of drug A (orange curve), n = 5. (B) Current density of INa at −20 mV, n = 5. (C) Influence of different concentrations of drug A on action potential

We used whole‐cell patch clamp to characterize the electrophysiological properties of the isolated cardiomyocytes, including action potential (Figure 2A) and the Ito current (Figure 2B) and L‐type Ca current (Figure 2C). The stimulus protocols are shown on the top right corner of the chart and the procedure. We further tested the effects of some drugs on the sodium current (INa) of these isolated cardiomyocytes. As shown in Figure 2D, INa from single myocytes under voltage‐clamp mode was readily evoked in all cells tested. We found increased INa in cardiomyocytes with drug A (Figure 2E) and showed the influence of drug A on the I‐V curve (Figure 3A) and the current density of INa at −20 mV (Figure 3B).

We further recorded the effects of drug treatment on action potentials of isolated cardiomyocytes. We found that the action potentials of cardiomyocytes were prolonged by drug treatment. Increasing the drug concentrations prolonged the action potential duration of the cardiomyocytes. After washing out the drug, the action potential duration was restored (Figure 3C).

### 3.3 The adult mouse heart is amenable to epicardial activation mapping technique

**FIGURE 4:**
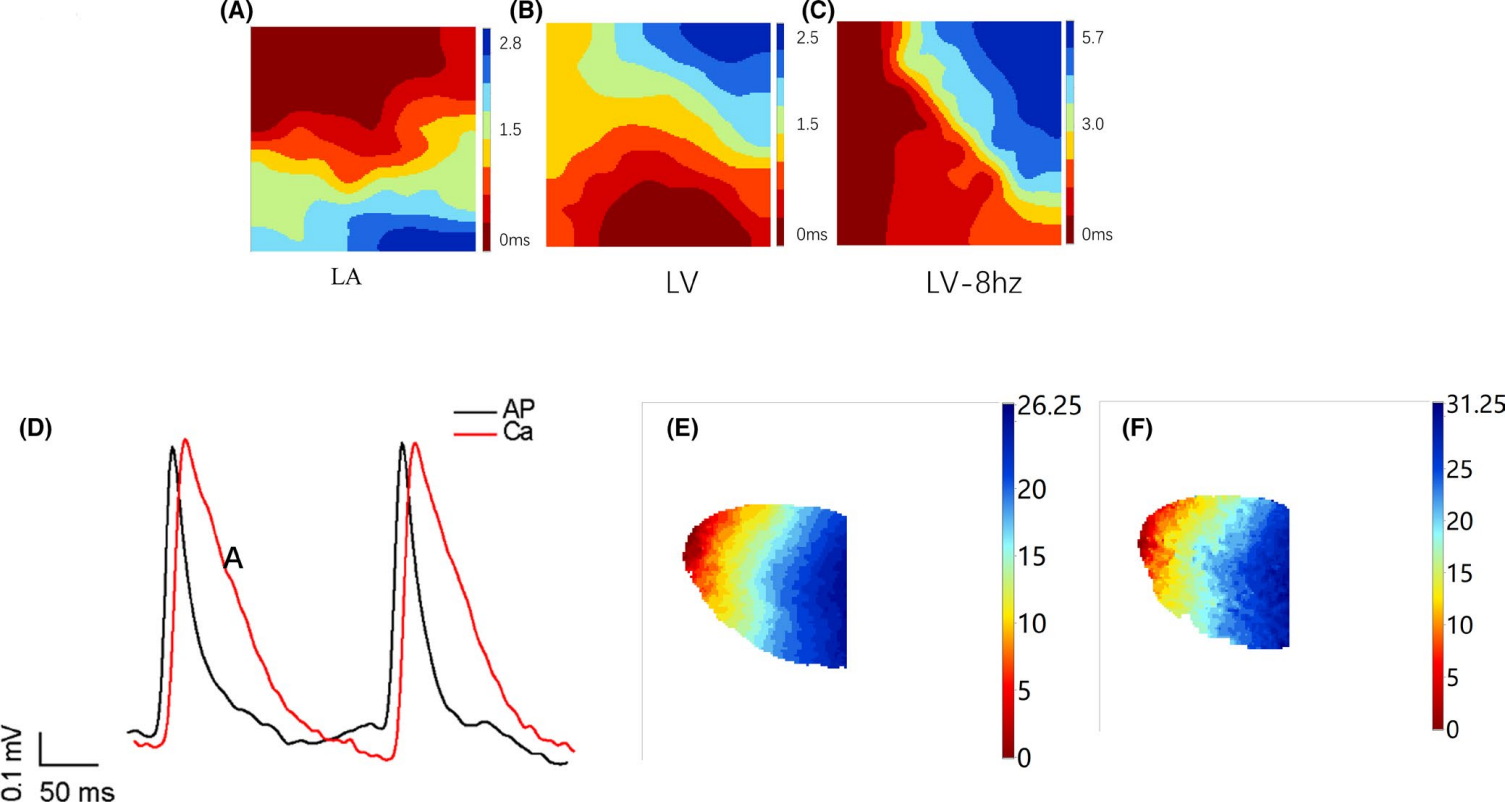
Electrophysiological epicardial activation mapping. (A) LA: representative activation maps of left atrium. (B) LV: representative activation maps of left ventricle. (C) LV‐8hz: representative activation maps of left ventricle by 8 Hz stimulation. Red indicates the first excited place, and blue indicates the last excited place; the direction of conduction is from red to blue. (D) Signals of APD_90_ (APD at 90% repolarization) and CAD_90_ (changes in intracellular calcium). *Scale bar, 50 ms. (E) Representative activation maps of action potential conduction with 5 Hz stimulation. (F) Representative activation maps of calcium with 5 Hz stimulation in the presence of different concentrations of drug A. Ruler: red indicates the first excited place, and blue indicates the last excited place; the conduction direction arrives from red to blue

To compare baseline electrical properties, a pacing electrode was positioned on the base of the left ventricular epicardium, that is left atrial sinus conduction (Figure 4A), left ventricular sinus conduction (Figure 4B) and left ventricular epicardial conduction, with 8 Hz stimulation (Figure 4C). To identify additional mechanisms underlying changes in cardiac electrophysiology, action potential duration (ADP) at 90% repolarization (APD_90_) was assessed with 5 Hz stimulation [heart rate, 400 beats per minute (bpm)] (Figure 4D). We also examined CaD_90_ (intracellular Ca2+ handling) at pacing frequencies (Figure 4D). Signals of APD_90_ and CaD_90_ in normal mouse hearts were recorded during Langendorff perfusion. Furthermore, we examined activation maps of action potentials and intracellular calcium levels using 5 Hz stimulation of the mouse hearts (Figure 4E and F).

## 4 DISCUSSION

Isolated adult mouse cardiomyocytes are a valuable model to study cardiomyocyte pathobiology and physiology, including calcium handling, contractility, bioenergetics, electrophysiology signalling, drug screening and apoptosis.^11^, ^12^, ^13^, ^14^ However, the traditional approaches to isolate mouse cardiomyocytes are often constrained by technical difficulties, leading to low yields of viable adult cardiomyocytes. In this study, we present a simplified method to isolate viable, calcium‐tolerant cardiomyocytes from adult mouse hearts using retrograde intubation through the abdominal aorta. Yields of total viable cardiomyocytes were in accordance with those previously reported.^15^, ^16^ Furthermore, we simplified the intubation method of epicardial activation mapping, greatly reducing the technical difficulty and increasing the probability of success. Although a simplified Langendorff‐free method^5^ has been used to concomitantly isolate the viable cardiac myocytes from adult mouse hearts, it requires more enzymes and manual and continuous operation.

The traditional method of Langendorff retrograde aortic perfusion includes the following steps: (a) open the mouse chest and dissect out the heart; (b) clean the tissues surrounding the aorta in a calcium‐free tyrode buffer; and (c) retrograde insert the aorta into the Langendorff perfusion device. This procedure is difficult, however, because (a) the mouse aorta is too small to be easily identified with the naked eye, and it is prone to pruning mistakes; (b) when hanging the heart, it is very difficult to lift the aortic opening with forceps, consequently resulting in high probability of aorta tearing; and (c) connecting the aorta to the Langendorff device is also a fragile operation that requires a stereoscope. It is also well known that the survival rate and activity of isolated cardiomyocytes are greatly influenced by operation time. The shorter the operation duration, the higher the cardiomyocyte survival. The traditional isolation approach is time‐consuming and requires a stereoscope, thus requiring highly trained operators.

Our optimized method includes the following steps: (a) open the abdomen and chest after anaesthesia; (b) retrograde puncture through the abdominal aorta and fixation; and (c) remove the blood vessels above the abdominal aorta and heart, and immediately hang the heart on the Langendorff perfusion device. Since the puncture occurs *in vivo*, the steps of pruning, gripping the aorta, and hanging the heart are omitted, which greatly reduces the technical difficulty of operation and shortens the operation time. Moreover, this procedure does not require a stereoscope, and the high survival rate of isolated cardiomyocytes allows for subsequent *in vitro* experiments.

Some experimental techniques, such as the working‐heart and mapping with the Langendorff perfusion device, which can be used to measure various cardiac functional parameters including ventricular pressure, electric conduction and intracellular calcium, are also hampered by the difficulty of aorta retrograde perfusion. Therefore, these experiments currently have not been widely used in the field. Our method simplified the operation process and shortened the operation time, which favours the wide application of these techniques.

In summary, we developed a novel, simplified approach to isolate adult mouse cardiomyocytes and map epicardial activation. We also demonstrated that mouse cardiomyocytes isolated using our method are suitable for drug screening and other *in vitro* experiments. Hence, our method can be used extensively in the field of the cardiac research.

Our study has some limitations that should be noted. The operator must be familiar with the anatomy of the mouse aorta and heart. In addition, the heart is directly transferred from the mouse chest cavity to the perfusion device. As a result, there is a potential risk of contamination in the isolated cardiomyocytes, similar to what occurs with other methods.

## CONFLICT OF INTEREST

The authors declare no potential conflicts of interest with respect to the research, authorship and/or publication of this article.

## AUTHOR CONTRIBUTION

Ziguan Zhang: Conceptualization (equal); Data curation (equal); Formal analysis (equal); Writing‐original draft (equal). Wuyang Zheng: Conceptualization (equal); Data curation (equal); Formal analysis (equal); Methodology (equal). Dehua He: Conceptualization (equal); Data curation (equal); Formal analysis (equal); Software (equal). Zichao Hu: Conceptualization (equal); Data curation (equal); Formal analysis (equal); Methodology (equal); Writing‐original draft (equal). Qiang Xie: Conceptualization (equal); Data curation (equal); Formal analysis (equal); Resources (equal). Meirong Huang: Conceptualization (equal); Data curation (equal); Formal analysis (equal). Weihua Li: Conceptualization (equal); Data curation (equal); Formal analysis (equal); Writing‐original draft (equal). Zhengrong Huang: Conceptualization (equal); Data curation (equal); Formal analysis (equal); Writing‐original draft (equal); Writing‐review & editing (equal). All authors participated in the design of the study, analysis and interpretation of the data, and review of the manuscript and approved the submitted version.

## ETHICS STATEMENT

## Supporting information

Figure S1‐S2

Video S1

## Data Availability

The raw data supporting the conclusions of this article will be made available by the authors, without undue reservation.
