# Supplementary material for: An improved procedure for isolating adult mouse cardiomyocytes for epicardial activation mapping
Source: J Cell Mol Med. 2021 Nov 10;25(24):11257–63. doi: 10.1111/jcmm.17049 (PMC8650026; doi:10.1111/jcmm.17049)
Supplement: Supplementary file 1 — Figure S1‐S2 [file JCMM-25-11257-s001.pdf]

Supplemental figure 1: Procedure of mouse cardiomyocyte isolation

A. Puncture the abdominal aorta with an indwelling needle until to the level of the heart root.
B. Tie the suture line.
C. The heart was quickly inserted into the Langendorff system.

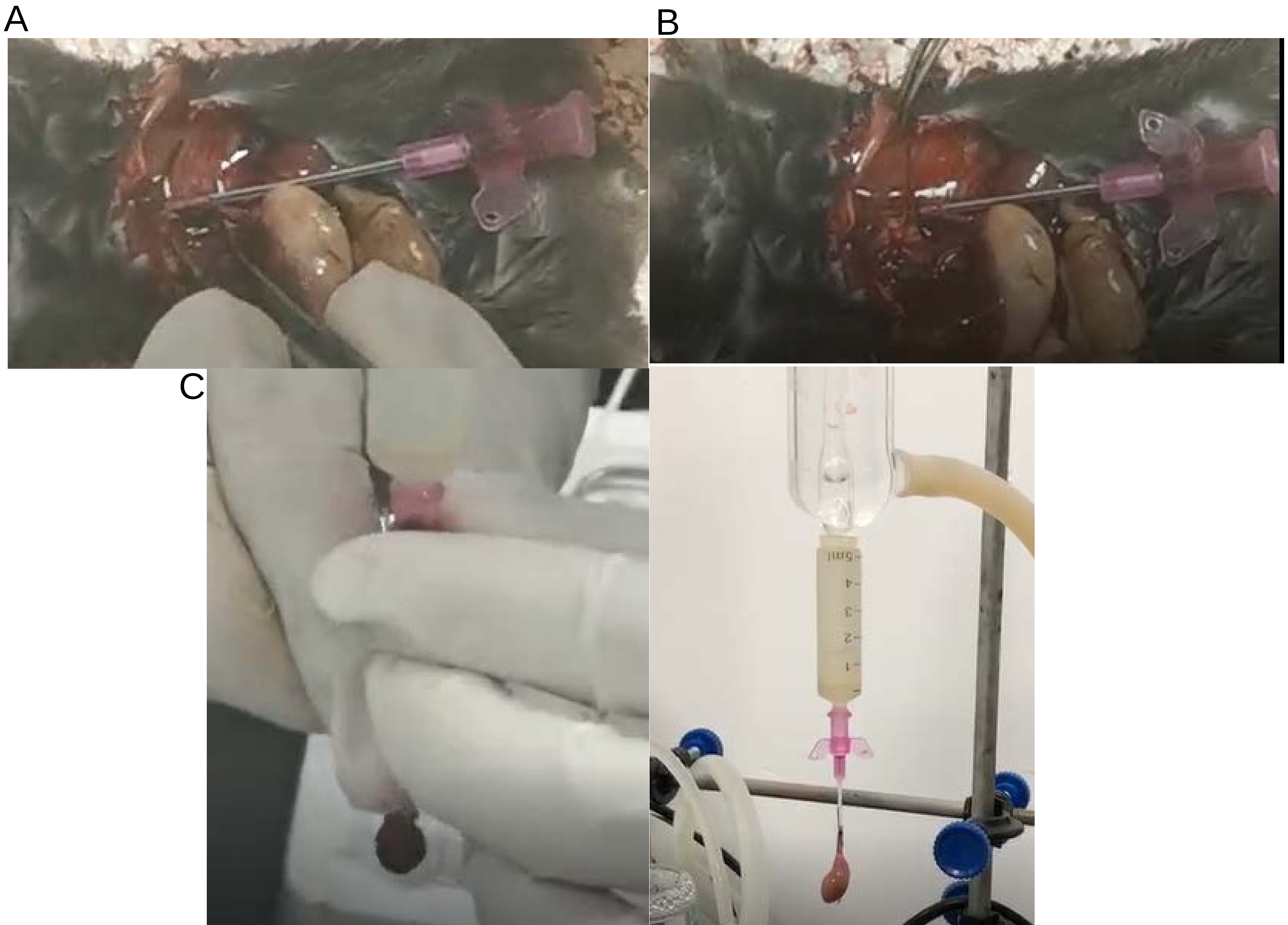

Supplemental figure 2. Characterization of isolated cardiomyocytes

A&B. Represent Isolated cardiomyocytes were cultured for 24h.

C. Representation of Isolated cardiomyocytes transfected by adenovirus with GFP.

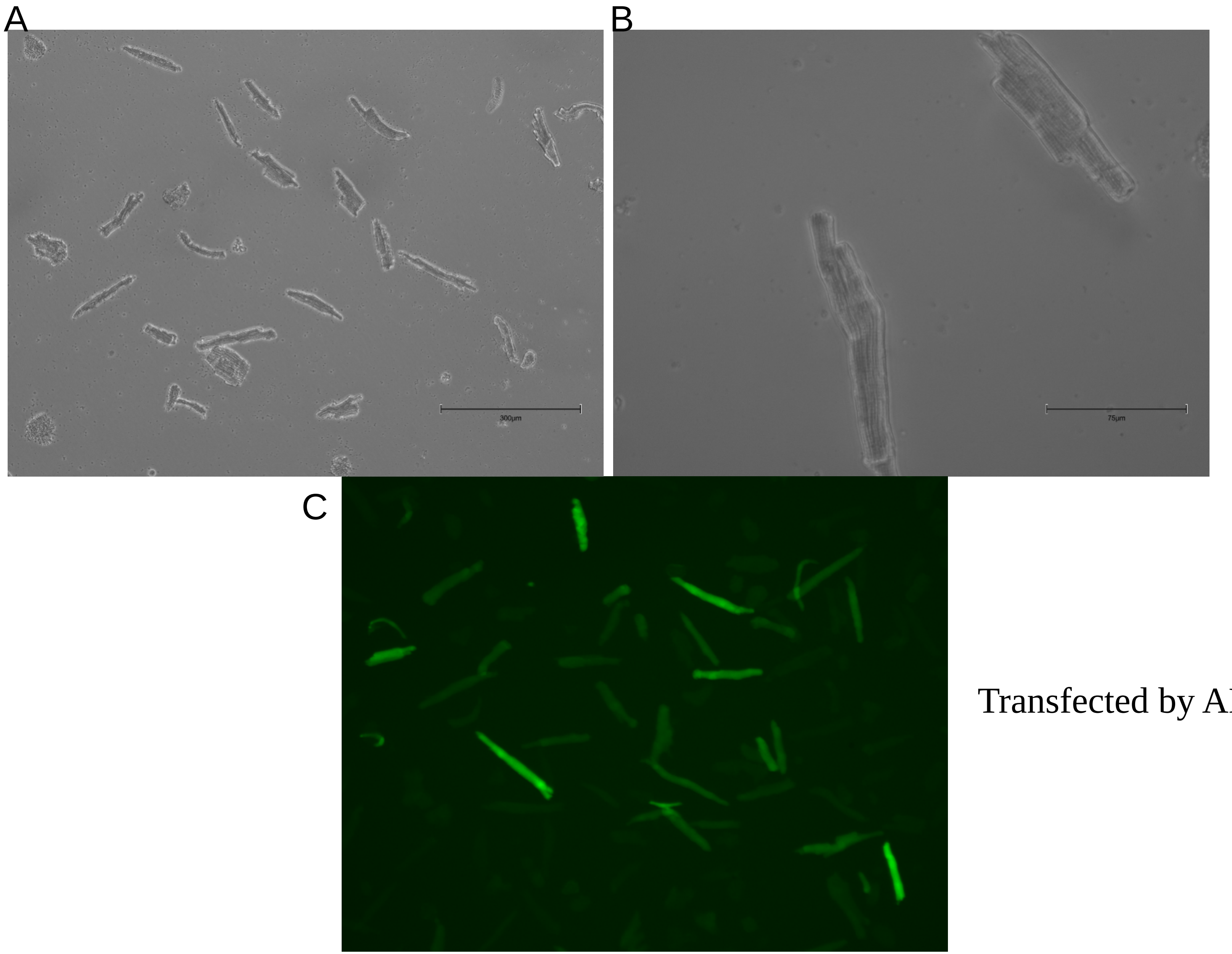

Culture for 24h

Transfected by ADV with GFP
